# Knowledge and attitudes on sexual and reproductive health among adolescents and youths in Refugee Settlements: A case of Palorinya, Uganda

**DOI:** 10.4314/ahs.v25i4.11

**Published:** 2025-12

**Authors:** Cissie Namanda, Mary Nakafeero, Claire Biribawa, Nazarius Mbona Tumwesigye, James Muhumuza, Muzamiru Tumwine, Charles Luwaga, Russell Dowling, Moses Otai

**Affiliations:** 1 Makerere University School of Public Health; 2 ChildFund Uganda; 3 ChildFund International

**Keywords:** Sexual and Reproductive Health, Knowledge, Attitude, Adolescents, Youth, Refugees

## Abstract

Sexual and reproductive health (SRH) of refugee adolescents and youths is a neglected area with high unmet needs. This paper presents the levels and factors associated with Knowledge and attitudes towards SRH services in the same population in Palorinya settlement, Uganda.

The cross-sectional study utilized proportional stratified sampling techniques to generate data from 801 participants. Knowledge of SRH focused on family planning, sexually transmitted infections, and menstruation. Multivariable modified Poisson and logistic regression analyses were conducted to identify factors associated with knowledge and attitudes regarding SRH.

Overall, knowledge of FP and STIs was high, at 75% and 94.5%, respectively, but knowledge of menstruation was low, at 6.5%. Having knowledge of FP was associated with age 16-19 years (APR 1.75, 95% CI=1.55-1.98), age 20-24 years (APR 1.86; 95% CI=1.63-2.12), having a monthly income (>=50,000 (APR=1.33; 95%CI (1.12-1.59)] and being married/in-union (APR=1.09, 95%CI:1.02-1.1.16). Nearly the same pattern was observed for knowledge of STIs, menstruation, and positive attitudes toward SRH.

The level of knowledge of SRH on STIs and FP was higher than what was reported in other studies among young refugees in Africa. Knowledge on SRH increased with education; thus, increasing opportunities for higher education level is likely to improve knowledge on SRH, though this is subject to further investigations

## Introduction

By the end of 2019, the United Nations High Commissioner for Refugees (UNHCR) had recorded a global population of forced displacement at 70.8 million, with 36.6% (25.9 million persons) officially classified as refugees and over half under the age of 18^1^. As of 2020, Uganda was home to over 1 million refugees and asylum seekers, ranking it among the world's largest refugee-hosting countries and the largest in Africa^1^. The refugees in Uganda are hosted in settlements spread throughout the whole country, with approximately 61.7% from South Sudan and 29.3% from the Democratic Republic of Congo (DRC). Women and children account for 81% of the refugees from the DRC^1^.

Uganda has one of the youngest populations, with 42.2%of its population being below the age of 15 years and 25% being adolescents^1^. Adolescents range in age from 10 to 19 years, whereas youths range from 15 to 24 years^2^. Adolescent stages involve physical, cognitive, behavioral, and psychosocial changes. This is a period where people get to experiment with everything since they feel independent, which increases their risk of health problems^3^. In Uganda, teenage pregnancy is 25%, and 16.9 is the median age at first sex^4^. Among the annual new HIV infections, 39% are among young people^5^.

Despite these challenges, sexual and reproductive health (SRH) remains overlooked, under-resourced, and lacking in the country^6^-^8^. Adolescents and youths face difficulty accessing reproductive health services such as voluntary, informed, and affordable family planning services, prenatal care, safe motherhood services, assisted childbirth from a skilled birth attendant, and the prevention and treatment of sexually transmitted infections (STIs), including HIV^9^. The prevention and treatment of violence against women and girls, safe and accessible post-abortion care, and sexual health information, education, and counseling are also lacking.

The situation is even worse in humanitarian settlements where there is increased vulnerability to sexually transmitted infections and unwanted pregnancies^10^,^11^. A study conducted among refugee adolescent girls in Western Uganda reported that 11% had ever had sex^12^. In Bidibidi, a refugee settlement in the West Nile region in Uganda, 38% of adolescents reported having ever had sex^9^. A review of reports from different countries worldwide identified geographical accessibility, challenges with service availability, knowledge of services, and provider-client communication as some of the limitations to accessing SRH among refugees^11^. Other limitations include disruptions to family and social structures, poor living conditions, inadequate sanitation, and limited access to health services associated with conflict and displacement^13^. Additionally, the stigma associated with sexual activity during adolescence has been highlighted as another barrier to accessing SRH services^6^. It has been further underscored that forced migration increases the vulnerability of adolescents and youths, compromising their power to negotiate and make decisions related to sexual relationships^6^,^14^. Maternal health is one of the four key clusters of primary healthcare. This can be achieved through sexual and reproductive health and its rights. To accomplish these, strategies should take into consideration a safe sex life, the right to choose when to give birth, and access to SRH information. Thus, refugees should have access to correct information as well as safe, effective, and voluntary acceptable and affordable family planning methods and sexually transmitted preventive options. In addition, it should focus on safe motherhood services, prevention of sexually transmitted diseases, and sexual violence^15^.

There is limited information available on SRH among adolescents and youths in refugee settings^16^. To improve sexual and reproductive health (SRH) for adolescents and youths, we need to understand current knowledge and attitudes to identify tailored interventions suitable for this population in the context of conflict and displacement.

The available literature tends to report on the SRH needs of young people to improve the availability of family planning in refugee settlements, address language barriers, and adequately strengthen the inadequate capacity of healthcare workers^17^-^19^. A study by Ivanova, Rai, and Kemigisha^11^ in one of Uganda's refugee settlements found that health workers are not youth-friendly; in most cases, they judge and condemn adolescents for irresponsible behavior^6^.

A study was conducted to fill the knowledge gap and provide an overview of the situation regarding sexual and reproductive health knowledge, attitudes, utilization of, and access to services among adolescents and youths living in Palorinya Refugee Settlement and host communities in Obongi District, Uganda. This paper presents the findings on knowledge and attitude from that study.

## Methods

### Study setting

Palorinya refugee settlement was established in December 2016 and is located in Uganda's Obongi District in the West Nile region. The settlement currently hosts mostly South Sudanese refugees fleeing from the violent conflict in South Sudan. In 2023, the Palorinya settlement was estimated to host nearly 130,000 South Sudanese refugees, with a total surface area of 37.58 square kilometers^17^. The youths (adolescents and youths aged 15 – 24 years) comprise 25% (32,535) of the total refugee population and face most of the SRH challenges.

### Study design and sampling

This cross-sectional design study involved collecting data through a household survey. The study targeted refugee adolescents aged 13-19 years and youths aged 20-24 years in both refugee and host communities in the Palorinya refugee settlement. Proportionate stratified sampling was used to select a commensurate number of households from each of the five zones of the settlement, as shown in Table 1 below. The selection criteria for respondents included gender, age (13-19 years old and 20-24 years old), willingness to participate in the study, informed consent from a parent or caregiver, and permission for minors. The study reached 801 households (participants), including 592 adolescents and 209 youths.

**Table 1:** Distribution of adolescents by socio-economic characteristics of adolescents and youths in refugee

Characteristic	Total (N=801)	Col. Percentage (%)
*Age*		
13-15	313	39.1
16-19	273	34.8
20-24	209	26.1
*Gender*		
Male	263	32.3
Female	538	67.2
*Highest level of education*		
None	58	8.5
Primary	508	63.4
Secondary/Tertiary	225	28.1
*Current occupation*		
In School	556	69.4
Employed	51	6.4
Apprenticeship	24	3.0
Others	170	21.2
*Monthly income (Ugx)*		
No income	74	9.2
< 50,000	603	82.9
At least 50,000	124	17.0
*Marital status*		
Not in union	633	79.9
In union	151	20.1
*Religious affiliation*		
Catholic	297	37.1
Anglican	138	17.2
Pentecostal/Born Again/PAG	313	39.8
Other religion	47	5.9
*Country of Origin*		
South Sudan	740	92.4
Uganda	61	7.6

### Data collection

For the quantitative data collection, smart mobile phones were programmed using the XML file format with the ODK (Open Data Kit) software and utilized for the exercise. The data were entered offline and only connected to the internet for uploading. The questionnaire was designed using validated tools: the Reproductive Health Assessment Toolkit for Conflict-Affected Women by the CDC^21^ and the Adolescent Sexual and Reproductive Health Toolkit for Humanitarian Settings by UNFPA and Save the Children^20^.

### Measurement of variables

#### Dependent variables

Knowledge of sexual reproductive health was measured by three variables: knowledge of family planning, knowledge of sexually transmitted infections, and knowledge of menstruation. For a respondent to be knowledgeable about family planning (FP), they should have heard about FP and a method of FP or should have possessed knowledge on the appropriate age for marriage. For someone to be knowledgeable about STIs, they should have known 2 out of 3 STIs, how to prevent STIs, and how HIV is transmitted. Knowledge of menstruation was measured by knowing the safe days during the menstruation cycle. To measure attitude variables, the study included 14 statements that asked participants to agree, disagree, or to respond ‘don't know.’ A positive attitude on SRH was defined as those who correctly responded to the 14 attitude questions. In contrast, those who had provided incorrect responses or did not know were taken to have negative attitudes. The constituting variables were summed up and converted into percentages. According to Bloom's cutoff points, those who scored 60% and above were categorized as having a positive attitude. In contrast, those who scored below 60% were classified as having a negative attitude (ref).

### Data management and analysis

The data sets were password-protected, and after data collection, they were exported to STATA V15. A cleaning exercise followed to ensure the data were valid and consistent. Following this, an analysis was conducted using STATA version 15.0.

Frequencies and corresponding percentages were obtained to summarize the joint distribution of the background characteristics and measures of reproductive health knowledge and attitudes. To identify factors associated with knowledge of FP, knowledge of STIs, and positive attitude towards SRH, we used modified Poisson regression model which approximates risk/relative ratios better than the odds ratios from a binary logistic regression model (https://pmc.ncbi.nlm.nih.gov/articles/PMC9993252/#R17) given the high prevalence of these outcomes. Logistic regression analysis was used to investigate the associations between knowledge of menstruation and the exposure variables, given the low prevalence of this outcome. Robust standard errors obtained under the Modified Poisson regression also adjusted for clustering. Collinearity was ruled out using variance inflation factors (VIF) which ranged from 1.02 to 2.08. Variables that were significant at the 0.2 level at bivariable analysis were considered for the multivariable model. Backward model building was subsequently employed for each outcome variable in order to arrive at the final set of variables in a parsimonious model. The sparse counts of respondents aged 13 to 15 resulted into relatively large odds ratios with wide confidence intervals for the age variable when analysing knowledge of indicating instability in the parameter estimates. Age was therefore excluded from the multivariable analysis.. The goodness of fit of the statistical models was assessed using the Hosmer-Lemeshow test. The final multivariable model results presented in Tables 3 and 4 had p-values (FP = 0.8322, STIs=0.9988, menstruation=0.8578, attitude=0.8685), indicating that the models fitted the data relatively well. Prevalence and odds ratios are presented with their corresponding 95% confidence intervals.

## Ethical considerations

Ethical clearance was obtained from the Institutional Review Board (IRB) of Makerere University, the School of Public Health Research Ethics Committee (Mak-SPH-REC), and subsequently from the Uganda National Council for Science and Technology (UNCST), the government body responsible for approving all research projects in the country. This enabled the research team to seek ethical clearance for the assessment from the Office of the Prime Minister (OPM) Representative at the settlement level—the Settlement Commandant. At the zonal level within the settlement, permission to conduct the assessment was obtained from the chairpersons of the zonal Refugee Welfare Committee (RWC). No individual identifier information was collected on the data collection forms, and all data collection staff signed a data confidentiality form before conducting fieldwork.

Before enrolment in the assessment, all participants aged 18 and above provided written informed consent. For participants under 18 years of age, informed consent was obtained from their parents, guardians, or caregivers, followed by their assent.

## Results

### Social demographic characteristics of participants

Eight hundred and one adolescents and youths participated in this study. The majority (67%) (538/801) were females, and 74% (592/801) were adolescents aged between 13 and 19 years. The highest number of participants were of the Kuku tribe (70%) (562/801), nearly all of whom (92%) (740/801) are South Sudanese. Less than a third (28.1%) (225/801) had secondary or tertiary as their highest level of education, while the majority (63%) (508/801) had completed only primary-level education, with almost 10% (68/801) having no formal education. Most of these adolescents and youths were still in school (69%, 556/801) and were, therefore, single or unmarried (74%, 593/801). We noted that those who were married (61%, 125/206) got married between the ages of 16 and 19 as shown in table 1.

### Knowledge of Sexual Reproductive Health among adolescents and youths in refugee communities

Knowledge of SRH was measured by three items: Knowledge of FP, knowledge of STIs, and knowledge of menstruation. Overall, Knowledge scores were highest for knowledge about STIs (94.5%), followed by knowledge of FP (75%). Knowledge of menstruation was very low (6.5%). About half (48.6%) of the adolescents aged 13-15 years had knowledge of FP, whereas nearly all (98.6%) youths aged 20-24 years were knowledgeable about FP. Knowledge of FP among those in school (68.2%) was less than those involved in other occupation categories (90-98%). For marital status, almost all refugees in the Union (98.1%) were knowledgeable about FP. The respondents from South Sudan had more knowledge of FP (76.1%) compared to the knowledge level of 68.9% among Ugandans living in the refugee communities. Knowledge of STIs was very high (over 88%) for all variable categories. There was not much variation in the studied characteristics. Knowledge of menstruation was minimal across all the studied characteristics. Among those with secondary education, only 14% had knowledge of menstruation, whereas other categories of this characteristic scored below 10%. Knowledge of menstruation for occupation was lowest for those employed (3.9%) and in school (4.5%) compared to those in apprenticeship (16.7%) as indicated in table 2.

**Table 2:** Knowledge of SRH as measured by Knowledge of FP, knowledge of STIs, and knowledge of menstruation

Knowledge of FP	Knowledge of STIs	Knowledge of Menstruation			
Characteristics		Total(N)	N.(row%)	N.(row%)	N.(row%)
**Overall**		801	605(75.5)	756(94.4)	52(6.5)
**Age**					
	13-15	313	152(48.6)	276(88.2)	4(1.3)
	16-19	279	247(88.5)	273(97.8)	19(6.8)
	20-24	209	206(98.6)	207(99)	29(13.9)
**Education**					
	None	68	58(85.3)	61(89.7)	6(8.8)
	Primary	508	331(65.2)	471(92.7)	18(3.5)
	Secondary/Tertiary	225	220(976.8)	(224(99.5)	28(12.4)
**Occupation**					
	In School	556	379(68.2)	520(93.5)	25(4.5)
	Employed	51	50(98.0)	51(100)	2(3.9)
	Apprenticeship	24	23(95.8)	24(100)	4(16.7)
	Others	170	153(90.0)	161(94.7)	21(12.4)
**Monthly income**					
	No income	74	42(56.8)	73(98.6)	-
	Less than 50,000	603	442(73.3)	560(92.9)	40(6.6)
	At least 50,000	124	121(97.6)	123(99.2)	12(9.7)
**Marital status**					
	Not in union	639	446(69.8)	596(93.3)	33(5.2)
	In union	162	159(98.1)	160(98.8)	19(11.7)
**Religion**					
	Catholic	297	224(75.4)	284(95.6)	16(5.4)
	Anglican	138	111(80.4)	135(97.8)	15(10.9)
	Pentecostal/Born Again/PAG	319	237(74.3)	294(92.2)	14(4.4)
	Other religion	47	33(70.2)	43(91.5)	7(14.9)
**Country of origin**					
	South Sudan	740	563(76.1)	700(94.6)	50(6.8)
	Uganda	61	42(68.9)	56(91.8)	2(3.3)

Key determinants of knowledge of FP and STIs were age, education, occupation, income, and marital status, while those for menstruation were age, gender, education, occupation, marital status, and religion as ported in Table 3.

**Table 3:** Bivariable analysis of factors associated with knowledge of sexual reproductive health

Background characteristics		Knowledge of FP	Knowledge of STIs	Knowledge of Menstruation
		Crude PR (95%CI)	Crude PR(95%CI)	Crude OR (95%CI)
**Age**				
	13-15	1.00	1.00	1.00
	16-19	1.82(1.61-2.06)***	1.11(1.05-1.16)***	5.65(1.9-16.81)**
	20-24	2.03(1.81-2.28)***	1.13(1.08-1.18)***	12.45(4.3-36.00)***
**Gender**				
	Male	1.00	1.00	1.00
	Female	0.97(0.89-1.05)	0.99(0.96-1.03)	1.89(0.95-3.74)*
**Education**				
	None	1.00	1.00	1.00
	Primary	0.76(0.68-0.86)***	1.04(0.95-1.14)	0.40(0.17-0.98)**
	Secondary or tertiary[WU1]	1.13(1.02-1.25)**	1.13(1.03-1.23)***	1.4(0.61-3.37)
**Occupation**				
	In School	1.00	1.00	1.00
	Employed	1.44(1.34-1.54)***	1.08(1.05-1.10)***	0.87(0.20-3.77)
	Apprenticeship	1.41(1.27-1.56)***	1.08(1.05-1.10)***	4.25(1.35-13.37)**
	Others	1.32(1.22-1.42)***	1.01(0.96-1.05)	2.99(1.63-5.50)
**Monthly income (Ugx)**				
	No income	1.00	1.00	
	Less than 50,000	1.29(1.05-1.58)**	0.93(0.90-0.97)***	-
	At least 50,000	1.72(1.41-2.10)***	1.01(0.97-1.04)	-
**Marital status**				
	Not in union	1.00	1.00	1.00
	In union	1.41(1.33-1.49)***	1.07(1.04-1.09)***	2.44(1.35-4.42)**
**Religion**				
	Catholic	1.00	1.00	1.00
	Anglican	1.07(0.96-1.18)	1.03(0.99-1.07)	2.14(1.03-4.47)**
	Pentecostal/Born Again/PAG	0.99(0.89-1.08)	0.96(0.92-1.01)*	0.81(0.39-1.68)
	Other (specify)	0.93(0.76-1.13)	0.94(0.85-1.04)	3.07(1.19-7.94)**
**Country of origin**				
	South Sudan	1.00	1.00	1.00
	Uganda	0.9(0.76-1.08) 0.98(0.9-1.06)		0.47(0.11-1.97)

*** p<0.01

** p<0.05

* p<0.1

PR=Prevalence ratio, OR=Odds ratio

After multivariable analysis, adolescents who were aged 16-19 years and youths 20- 24 years had almost two times the prevalence of knowledge of FP (APR 1.75, 95% CI=1.55- 1.98) and (APR 1.86, 95% CI=1.63- 2.12) compared to younger adolescents of age 13- 15 years. Participants with primary education had a 12% lower prevalence of being knowledgeable about FP (APR 0.88, 95% CI= 0.79-0.98) than those without education. Adolescents and youths who earned less than Ugx 50,000 or at least Ugx 50,000 had a prevalence of 25% (APR 1.25; 95% CI =1.07-1.48) or 33% (AOR 1.33; 95% CI=1.12- 1.59) more prevalence, respectively, compared to those who did not have any income. Ugandan adolescents and youths had a 15% lower prevalence of knowledge in FP (APR 0.85; 95% CI = 0.74-0.98) compared to those from South Sudan. There was marginal significance in the knowledge of FP across different categories of marital status and religion as indicated in table 4.

**Table 4:** Multivariable analysis of factors associated with sexual reproductive health

Knowledge of FP	Knowledge of STIs	Knowledge of Menstruation		
Characteristics		Adjusted PR(95%CI)	Adjusted PR(95%CI)	Adjusted OR (95%CI)
**Age**				
	13-15	1.00	1.00	-
	16-19	1.75(1.55-1.98)***	1.1(1.05-1.16)***	-
	20-24	1.86(1.63-2.12)***	1.12(1.06-1.19)***	-
**Gender**				
	Male	-	-	1.00
	Female	-	-	1.89(0.95-3.75)*
**Education**				
	None	1.00	1.00	-
	Primary	0.88(0.79-0.98)**	1.04(0.96-1.13)	-
	Secondary or tertiary	0.99(0.89-1.09)	1.06(0.99-1.14)*	-
**Occupation**				
	In School	1.00	1.00	1.00
	Employed	0.92(0.83-1.02)*	0.96(0.91-1.02)	0.89(0.19-4.08)
	Apprenticeship	0.97(0.84-1.11)	0.97(0.92-1.03)	4.4(1.41-13.73)**
	Others	0.9(0.81-1)*	0.93(0.87-1.01)*	3.28(1.74-6.20)***
**Monthly income (Ugx)**				
	No income	1.00	1.00	-
	Less than 50,000	1.25(1.07-1.48)***	0.94(0.90-0.97)***	-
	At least 50,000	1.33(1.12-1.59)***	0.97(0.92-1.02)	-
**Marital status**				
	Not in union	1.00	1.00	-
	In union	1.09(1.02-1.16)**	1.05(1.00-1.09)**	-
**Religion**				
	Catholic	-	-	1.00
	Anglican	-	-	2.18(1.04-4.58)**
	Pentecostal	-	-	0.67(0.32-1.43)
	Other (specify)	-	-	3.11(1.24-7.77)**
**Country of origin**				
	South Sudan	1.00	-	1.00
	Uganda	0.85(0.74-0.98)**	-	0.38(0.1-1.44)

*** p<0.01

** p<0.05

* p<0.1

PR=Prevalence ratio, OR=Odds ratio

Knowledge of STIs was 6% higher among participants who reported having secondary or tertiary education as their highest level of education (APR 1.06; 95% CI = 0.99-1.14) compared to those with no education. Knowledge of STIs was 11% higher among those aged 16-19 years (APR 1.1; 95% CI= 1.05-1.16) compared to adolescents 13-15 years. There was marginal significance for occupation, monthly income, and marital status.

The odds of knowledge of menstruation were almost two times higher among females (AOR 1.89; CI=0.95- 3.75) compared to males. Participants who were involved in the apprenticeship were four times (AOR 4.4; 95% CI=1.41-13.73) more likely to know about menstruation compared to those in school. Uganda participants had 62% lower odds of knowing about menstruation compared to refugees from South Sudan.

### Attitude on Sexual Reproductive Health among adolescents and youths in refugee communities

To assess attitudes toward SRH, participants were asked to either agree, disagree, or answer a statement. When the respondents were asked if there was no harm in having sexual intercourse below 18 years, 25% agreed, although 11% did not know whether it was okay or not to have sexual intercourse while below 18 years. More than half agreed that it was taboo to talk about sex in public, while 9.5% did not know. Equally important is that about 23% of the participants agreed that getting married before 18 years is harmless, and 18% did not think that having sex shows love for your partner or not. More than 18% of the participants also believe that when you have sex for the first time, you do not get HIV/AIDS, while others did not know (25%) as indicated in Table 5

**Table 5:** Attitudes of adolescents and youths towards SRH

Attitude and Perceptions questions	Agree		Disagree		Don't know	
	Freq	%	Freq	%	Freq	%
No harm in having sex before 18 years?	196	24.6	512	64.2	90	11.2
It is a taboo to talk about sex in public	492	61.5	232	29.0	76	9.5
No harm in getting pregnant before 18 years	184	23.0	534	66.8	82	10.2
There is no harm when girls abort	38	4.8	686	85.8	76	9.5
Getting married before 18 years is harmless	180	22.5	559	69.9	61	7.6
Access to contraceptives is easy	343	43.0	171	21.4	284	35.6
You do not get HIV/AIDS when you have sex the first time	141	17.6	461	57.6	198	24.8
I find the behaviour of Health workers towards adolescents seeking SRH services to be acceptable to me	535	66.9	73	9.1	192	24.0
All contraceptives are harmful to my health	136	17.0	377	47.1	287	35.9
Early childbearing is dangerous (Probe below 18 years of age)	709	88.9	55	6.9	34	4.3
Contraceptive usage prevents pregnancy	578	72.3	31	3.9	191	23.9

### Factors associated with the attitude of adolescents and youths toward SRH

From bivariate and multivariable analyses of adolescents' and youths' attitudes towards SRH, the prevalence of positive attitude was two times for adolescents aged 16-19years (APR 2.07; 95% CI=1.42- 3.01) and almost three times more for youths 20-24 years (APR 2.92; 95% CI=1.81- 4.71) compared to the adolescent aged 13-15years. Women were twice as likely to have a positive attitude (APR 1.97; 95% CI=1.43- 2.72) compared to men. Participants with secondary or tertiary education had almost twice the prevalence of positive attitudes compared to those with no education. Equally important were participants who reported having some income. Those who earned less than Ugx 50,000 and those who earned at least Ugx 50,000 were 6.4 and 4.8 times more likely to have positive attitudes, respectively, compared to those with no income as presented from Table 6.

**Table 6:** Attitude of adolescents and youths towards SRH and associated factors

Total	Positive attitude	Crude	Adjusted		
				n(Row%)	PR(95%CI)
**Overall**	N=801	449(56.1)			
**Age**	13-15	313	137(43.8)	1.00	1.00
	16-19	279	166(59.5)	1.36(1.16-1.59)***	2.07(1.42-3.01)***
	20-24		209	146(69.9)	1.6(1.37-1.86)***
**Gender**					
	Male	263	126(47.9)	1.00	1.00
	Female		538	323(60.0)	1.25(1.09-1.45)***
**Education**					
	None	68	35(51.5)	1.00	1.00
	Primary	508	261(51.4)	0.99(0.78-1.28)	1.45(0.82-2.55)
	Secondary or tertiary		225	153(68.0)	1.32(1.03-1.69)
**Occupation**					
	In School	556	289(52.0)	1.00	
	Employed	51	28(54.9)	1.06(0.81-1.37)	
	Apprenticeship	24	16(66.7)	1.28(0.96-1.72)	
	Others		170	116(68.2)	1.31(1.15-1.5)***
**Monthly income (Ugx)**					
	No income	74	15(20.3)	1.00	1.00
	Less than 50,000	603	356(59.0)	2.91(1.84-4.6)***	6.44(3.39-12.23)***
	At least 50,000		124	78(62.9)	3.1(1.94-4.97)***
**Marital status**					
	Not in union	639	343(53.7)	1.00	
	In union		162	106(65.4)	1.22(1.07-1.39)***
**Religion**					
	Catholic	297	156(52.5)	1.00	1.00
	Anglican	138	86(62.3)	1.19(1.00-1.40)**	1.79(1.12-2.85)**
	Pentecostal	319	181(56.7)	1.08(0.93-1.25)	1.22(0.86-1.72)
	Other (specify)		47	26(55.3)	1.05(0.8-1.39)
**Country of origin**					
	South Sudan	740	416(56.2)	1.00	
	Uganda	61	33(54.1)	0.96(0.76-1.22)	

*** p<0.01

** p<0.05

* p<0.1

PR=Prevalence ratio

## Discussion

The study found a high level of knowledge of FP and STI but a very low knowledge of Menstruation. This knowledge among adolescents and youths varied by age, gender, education level, marital status, and income, as reported in other studies^21^. Our findings indicate a higher level of knowledge of FP and STI compared to the 44% reported by Finlay et al. among SSA adolescent. The difference in the two studies may be explained by the different ages of the respondents. In our study, the sample consisted of individuals aged 13-24 years, whereas the study by Finlay et al. focused on adolescents aged 10-19 years^22^.

The majority of the participants had primary as their highest level of education; this group had a lower level of knowledge of FP. This will have an impact on their FP choices and fertility, leading to unplanned pregnancies^23^. Knowledge of SRH was generally lower among young adolescents compared to older age groups. This is a similar finding to what was reported in Ghana, where adolescents had little or no knowledge of reproductive health services.

The findings from our study of high levels of Knowledge on STIs is in line with a study among migrants and refugees from Sydney and Australia who reported high awareness of STIs^24^.

The majority of refugees lacked knowledge about menstruation. Similar findings have been reported in Bangladesh, where adolescent girls reported limited knowledge of menstruation^22^. However, our study has even much lower level compared to a report on SRH conducted across SSA. The level of knowledge about menstruation may be due to the difference in population, as our respondents were from refugee settings, which increased their vulnerability to limited knowledge^6^.

Knowledge on menstruation was slightly higher among females than males, which is similar to what was reported in Malaysia^25^. The difference may be explained by the fact that females experience menstruation and menstrual products as reported in the study conducted among young adults^26^.

Attitudes towards SRH increased with age, education, and income status, and were more prevalent among females. A study conducted among refugees in Guinea reported that women had fewer misconceptions about SRH concepts like bad touch. The same study reported that participants with some formal education were more likely to have a positive attitude toward SRH^27^.

## Conclusion

A high number of adolescents and youths living in refugee and refugee-hosting communities in Obongi District had high knowledge of FP, though they possessed limited knowledge of menstruation. Knowledge of SRH increased with age. Females were more knowledgeable about menstruation and had more positive attitudes to SRH. Ugandans who stayed within the refugee setting were less knowledgeable about SRH. Attitude improved with age, education level, and income status. We recommend including information on menstruation as part of SRH. Family planning and STIs should be introduced earlier, as young adolescents tend to have less knowledge of family planning.

The strength of the paper lies in its study of both adolescents and youths.

## Limitations

As this is a secondary data analysis, we did not have access to all the variables necessary to measure sexual reproductive health knowledge, and some variables were not measured appropriately. Some variable categories have sparse cells, resulting in prevalence or odds ratios with wide confidence intervals.

## Data Availability

The data set for this paper will be available on acceptance.
